# Changes in Teenagers’ Dietary Choices in Smart School Canteens: A Pre-Post Single-Arm (Quasi-Experimental) Study of a Digital Nudge Intervention

**DOI:** 10.3390/nu17172782

**Published:** 2025-08-27

**Authors:** Zuoyi Liang, Mingshi Hao, Rui Fan, Xuerui Wang, Wenli Zhu, Zhaofeng Zhang

**Affiliations:** 1 Department of Nutrition and Food Hygiene, School of Public Health, Peking University, Beijing 100191, China; liangzuoyi@bjmu.edu.cn (Z.L.); 15652916471@163.com (M.H.); fanruirf@bjmu.edu.cn (R.F.); zhangzhaofeng@bjmu.edu.cn (Z.Z.); 2 Public Nutrition and Development Center, National Development and Reform Commission, Beijing 100824, China; xuerui1984@pku.org.cn

**Keywords:** student meals, food choice, smart canteen, digital nudge

## Abstract

**Background/Objectives**: Adequate adolescent nutrition is vital for lifelong health, yet traditional school meal programs often emphasize processed foods. Digital nudges, subtle digital changes, may help promote healthier food choices. This study aimed to evaluate the impact of a digital nudge intervention in a smart school canteen on students’ food choices and nutrient intake over three months. **Methods**: A pre-post single-arm (quasi-experimental) study was conducted among 502 high school students (aged 15–17) in Shenyang, China. In August 2023, the school implemented a smart canteen with a mobile mini-program for meal pre-ordering. Embedded digital nudges included improved visibility of healthy options, nutritional information, and default settings favoring nutritious choices. Dietary intake was assessed using a 3-day 24 h dietary record and a food frequency questionnaire. Paired *t*-tests, Wilcoxon signed-rank, and chi-square tests were used for analysis. **Results**: Post-intervention, the weekly consumption frequency of coarse grains (*p* = 0.017), fruits (*p* < 0.001), seafood (*p* < 0.001), and soy products (*p* < 0.001) significantly increased, while sweets (*p* = 0.033), sugary drinks (*p* = 0.015), fast food, and eating out (both *p* < 0.001) decreased. Daily calcium intake rose from 683.00 mg to 804.11 mg (*p* < 0.1), and the proportion meeting recommendations increased from 39.3% to 50.9%. No significant change was observed in vitamin C intake (*p* = 0.192). **Conclusions:** The digital nudge intervention in the smart school canteen effectively improved students’ dietary choices, particularly increasing the consumption frequency of healthy foods and dietary calcium intake.

## 1. Introduction

Adequate nutritional support during childhood and adolescence is considered a key determinant of lifelong health and well-being [1]. The nutritional health of children is influenced by a confluence of factors at the family, school, and societal levels. The global health initiative of the World Health Organization (WHO) explicitly identified school as an ideal environment for enhancing students’ health and nutritional status, considering the significant portion of time students spend within this setting [2]. School meals are instrumental in shaping the overall dietary structure of students [3]. At the same time, studies have indicated that school meal programs have inadvertently emphasized the role of processed foods in improving nutrition, leading to increased consumption of sugar, unhealthy fats, and salt, while simultaneously limiting the intake of fruits and vegetables [4]. Children’s dietary intake and food selection are influenced by numerous factors, including the specific food environment in which they operate and from which they choose their meals. It is crucial to acknowledge the role of children’s decision-making in this context, but it is also essential to recognize that their choices are inherently limited by the food and beverage options available on the school canteen menu. To influence children’s food choices, nudges can be employed—these are subtle alterations to the physical and social environment that reshape the existing food choice architecture and the context in which decisions are made [4]. When healthier food options are accessible, nudges can effectively redirect school children’s food choices towards those that contribute to a nutritious diet. As such, they present a significant opportunity to promote healthy eating habits among schoolchildren. WHO recommends using nudge strategies such as modifying the physical environment, changing default options, and providing targeted information to encourage students to select healthier foods [2]. This strategy has shown promise in the school food environment for students [5,6]. Reviews showed that nudge interventions such as menu labeling, food placement, and prompts can positively influence students’ selection of healthy foods [7].

Digital technologies, ranging from social networking sites to online food delivery platforms, have seamlessly integrated into people’s daily lives across the globe. The digitalization of food environments has emerged as a critical public health issue, driven by technologies that improve food production, distribution, and accessibility—such as food delivery applications—and by the proliferation of health and nutrition information on social media. These developments have shaped the concept of the “digital food environment”, in which individuals actively engage and make dietary decisions. Within this context, the design of human–computer interaction interfaces is a key determinant influencing user decision-making processes [8]. By implementing nudge strategies in digital decision-making environments, such as guiding judgments and choices through user interface design, digital nudging can be effectively achieved [9].

In schools, a prominent example of digital nudging is the implementation of smart canteens. These canteens harness the power of big data, the Internet of Things, and artificial intelligence technologies to integrate digital solutions for ordering, dining, and organizational management. The objective is to elevate the dining experience, minimize waiting times, augment the nutritional quality of food, and offer personalized nutritional plans and data-driven support [10]. Research conducted in Australia has revealed that interventions utilizing smart canteens can significantly reduce the calories in student lunch orders by 567.25 KJ, saturated fat by 2.37 g, sodium by 227.56 mg, and sugar by 1.16 g [11]. Similarly, these interventions have been found to decrease the calories in student snack orders by 269.3 KJ, saturated fat by 1.1 g, and sodium by 128.6 mg [12]. Furthermore, in their 2020 Global School Feeding Report, the World Food Programme (WFP) mentioned that they have developed three digital initiatives, including an upgraded “menu planning” tool that employs algorithms to optimize school menus, thereby assisting schools in creating more cost-effective and nutritionally balanced meal plans [13].

Digital nudging plays a pivotal role in the advancement of smart canteens in schools [5], as it encourages students to opt for healthier food options. By influencing users’ emotional cognition and affective experiences, digital nudging can alter behavior patterns and heighten the preference for nutritious foods. Smart canteens employ various digital nudging strategies, such as emphasizing healthy foods on the ordering interface, optimizing recommendation positions, showcasing appealing food images, and offering nutritional feedback, to effectively prompt students to select healthier diets and enhance their overall dietary nutrition [5]. An experimental study on nudging in school online ordering demonstrated that providing nutritional feedback prior to ordering resulted in a decrease of 74.1 KJ in average total energy intake and a reduction of 0.4 g in average saturated fat intake. Additionally, the proportion of healthy food purchases increased by 3.8%, while the proportion of purchases of non-recommended foods decreased by 2.6% [14]. However, there is still a limited understanding of the scope and form of this rapidly evolving field. Furthermore, current food environment frameworks insufficiently acknowledge the role of digitalization in shaping food availability, acquisition, and consumption, as well as its potential implications for promoting healthy diets.

The establishment of smart school canteens in China began in 2020, following a policy initiative introduced by the Chinese Ministry of Education. This policy proposed integrating digitalization with school canteens to facilitate planned food procurement and offer personalized food supply and service [15]. However, there remains a lack of understanding regarding the scope and nature of smart school canteens in China. It also failed to fully consider its potential impact on students’ formation of healthy eating habits in the Chinese school environment. This study is to investigate how smart canteens in Shenyang, China—through the use of digital nudging strategies—affect students’ food choices and dietary quality. The main hypotheses of this study were as follows: (1) the digital nudge intervention would significantly improve the proportion of healthy food choices among students; and (2) the digital nudge intervention would significantly improve students’ nutrient intakes. This study seeks to provide empirical evidence to support the adoption of multi-strategy digital nudging interventions in school environments, thereby contributing to healthier food selections and improved nutritional outcomes for students.

## 2. Materials and Methods

### 2.1. Study Design and Participants

This is a pre-post single-arm (quasi-experimental) study conducted in a public senior high school situated in Shenyang, China. The baseline data were collected in August 2023, immediately before the launch of the smart canteen system. The follow-up data were collected three months later, in November 2023, after the system had been in operation for three months. This system presented a unique opportunity to improve students’ dietary choices through the application of a digital nudge intervention strategy. The outcomes were evaluated at baseline and three months after the consecutive semester of utilizing the ordering system.

In this study, relying on the smart school canteen system management enterprise, a senior high school with a smart canteen system in Shenyang City was selected as the investigation site. All students in the non-graduation grade and their main caregivers at the survey site were selected as the survey subjects, and the 10th and 11th grades (15–17 years old) of senior high school were selected according to the actual situation. On this basis, students and parents were convened as research subjects on a voluntary basis.

Based on a prior trial study examining the impact of school online ordering-based nudge intervention [7], the results indicated a 1.2-fold increase (equivalent to 3.8 percentage points) in the frequency of consuming healthy food. The formula utilized for sample size calculation is outlined below:

$$N\;=\;\frac{2({t_{\alpha /2}+t_{\beta })}^{2}\sigma^{2}}{d^{2}}$$

In the formula, N represents the sample size, *α* = 0.05, and *β* = 0.1. The calculated sample size was 355. Considering a 10% loss rate due to follow-up and a 20% uncompletion rate of investigations, the final sample size was adjusted to 493. Ultimately, informed written consent was voluntarily obtained from 774 student–caregiver pairs, yielding a response rate of 96.5%. The baseline survey encompassed a total of 638 participants, while the follow-up survey included 649 participants, with 519 overlapping between the two surveys. The number of valid samples that participated in both surveys with a completion rate exceeding 80% amounted to 502, surpassing the adjusted sample size of 493.

### 2.2. Digital Nudge Intervention Strategy of Online Ordering System in the Smart School Canteen

The smart canteen system in the school is mainly composed of several key components: user terminal, query terminal, guidance screens, meal pickup terminal, and a robust system backend. The user terminal, primarily accessible via a mobile application, facilitates school meal online ordering for students and their caregivers for the upcoming week while at home. The query terminal, strategically placed within the school canteen, enables students to check and modify their school meal orders as needed. The guidance screen efficiently directs students to their pre-assigned meal pickup counters, ensuring a smooth and orderly process. The meal pickup terminal, meanwhile, displays real-time meal ordering information for the canteen staff, facilitating efficient service. Lastly, the system backend serves as the central hub, allowing dietitians to access and analyze valuable data on students’ dietary intake. This comprehensive data set provides dietitians with deep insights into students’ eating habits and preferences.

**Figure 1:**
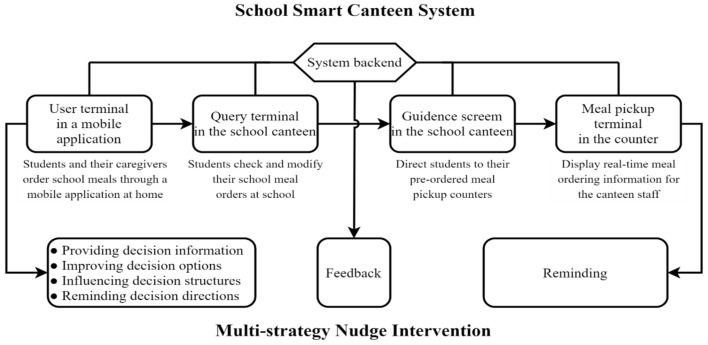
Digital nudge intervention strategy in the smart school canteen.

The smart canteen system incorporates a multi-strategy choice architecture intervention, designed to enhance healthy food choices among students. This intervention structures environments in ways that cue desirable and automatic selection of health items from the menu. Choice architecture strategies include providing nutrition labels at the point of purchase, positioning healthier items more prominently and easily accessible, and using prompts or automatic defaults to gently ‘nudge’ consumers towards healthier choices, as illustrated in Figure 1.

The online meal ordering section leverages various digital nudges to influence students’ choices:

Providing decision information: Each item on the online menu is labeled with simplified nutritional labeling information. A detailed description of the nutrition and health information, including recommended food categories and quantities based on the Chinese dietary guideline, labeling information analysis, and more, is also accessible.

Improving decision options: Healthier items are prominently featured on the menu (changing defaults). Esthetic and appealing food images are used in the meal ordering list and food detail pages to enhance the appeal of healthy options.

Influencing decision structures: Healthier food categories, such as whole grain, vegetables and fruits, aquatic products, dairy, and bean products, are prioritized and positioned first with enlarged entry points, to increase the visibility and accessibility of healthy options.

Reminding decision directions: Prior to confirming each order within the online ordering system, users receive feedback summarizing the nutritional content of the order from an AI dietitian. The AI dietitian also provides personalized advice to improve the menu based on individual health status and nutrient requirements. After completing the order, the AI dietitian reminds users about food choices on weekends to compensate for the school meals consumed during the weekdays.

In addition to these strategies, supplementary support is provided in other sections of the smart canteen system. At the meal pickup counter, canteen staff remind students to finish their dishes. Once a month, the system backend provides an overall menu analysis and feedback suggestions to students and their caregivers via the user terminal.

### 2.3. Investigation of Dietary Intake in School Meals

To assess the impact of digital nudge interventions on students’ dietary intakes, we collected 24 h dietary records for three days (two school days and one weekend) before and after the implementation of the school’s smart canteen system, spanning a period of three months after the implementation.

Data collection was streamlined through the use of an online platform called Boohee, which provided seamless integration and accessibility. Prior to the survey, field personnel distributed food scales to the participating students and conducted training sessions to ensure accurate recording of dietary intake. Throughout the recording period, participants were consistently reminded to diligently document their meals daily, thereby enhancing the integrity and reliability of the collected data.

Utilizing a comprehensive database sourced from the China Food Composition [16], the daily average intake of various nutrients, including carbohydrates, proteins, fats, calcium, iron, zinc, and vitamins A, B1, B2, and C, which ensured by accurate and reliable estimates of nutrient intake among the study population.

### 2.4. Investigation of Food Consumption Frequency

Addressing potential unhealthy eating behaviors, the frequency of food consumption was investigated using a self-administered questionnaire. The survey covered the frequency of intake across various food categories, including whole grains, vegetables, fruits, seafood, eggs, dairy products, soy products, breakfast, sweets, sugar drinks, fried foods, and fast food, over the past week. It also inquired about the timing of breakfast consumption and the location of meals, such as eating out. Participants were asked to report their frequency of consumption using categorical responses ranging from “None” to “Everyday” (“1–2 days”, “3–4 days”, “5–6 days”). The questionnaire items and response options were based on the relevant literature [17]. The questionnaire completion process was guided by the investigators in the classroom to ensure accuracy and completeness.

Additionally, we collected demographic data from the participants, including their gender, grade, household registration status, proportion of household food expenditure, principal caregiver, and the caregiver’s education level. This information was gathered to provide a comprehensive understanding of the participants’ background and potential influences on their food consumption habits.

### 2.5. Statistical Analysis

Data arrangement and statistical analyses were performed utilizing SPSS software (version 26.0).

The adequacy of food intake in school meals was assessed based on the Chinese nutrition guidelines for school meals (WS/T 554) [18]. The dietary nutrient intakes from school meals were compared to the Chinese Dietary Reference Intakes (DRIs 2023) [18]. Inadequate intake was identified when the consumption of calcium, iron, zinc, and vitamins A, B1, B2, and C fell below the estimated average requirement (EAR). Furthermore, the percentage of energy (%E) derived from protein, carbohydrate, and fat was compared to the acceptable macronutrient distribution ranges (AMDR).

In accordance with the Chinese Dietary Guideline, the food consumption frequency was categorized into “daily” and “≤6 days” for whole grains, vegetables, fruits, seafood, eggs, dairy products, soy products, and breakfast. For sweets, sugary drinks, fried food, fast food, and eating out, the categories were “weekly (at least once a week)” and “none”.

Descriptive statistics were employed to present the characteristics of the participants. Differences in food and nutrient intake between baseline and post-intervention were compared using independent-samples *t*-tests and Chi-square tests. For data with non-normal distribution, non-parametric test methods such as the Mann–Whitney U test were primarily used. The independent variable was the intervention condition (baseline vs. digital nudge intervention), and the dependent variables were the proportions of selected food categories (e.g., fruits, vegetables, sugar-sweetened beverages) and the nutrient intake levels (e.g., energy, protein, fat, calcium, vitamin C). Besides the conventional significance level of *p* < 0.05, we also reported marginally significant results when *p* < 0.1.

## 3. Results

### 3.1. Demographic Characteristics of Participant Students

Since the survey did not require mandatory completion for fields other than gender, there were some missing values. Of the participants, 278 out of 502 participants (55.4%) were in the 10th grade, 224 out of 502 participants (44.6%) were in the 11th grade, and the ages were mainly concentrated between 15 and 17 years old. Regarding the primary caregiver, 409 out of 502 participants (81.4%) were mothers, 76 out of 502 participants (15.1%) fathers, and 18 out of 502 participants (3.5%) others (mainly grandparents/grandmothers, other relatives/friends); for the educational level of the primary caregiver, 274 out of 502 participants (54.5%) had a high school education or below, and 228 out of 502 participants (228 out of 502 participants (45.5%)) had a college education or above. Further details are available in Table 1.

### 3.2. Comparison of Students’ Food Choice Before and After the Digital Nudge Intervention in Smart School Canteen

After the system was implemented, students reported higher daily consumption of coarse grains, fruits, seafood, and soy products, and lower consumption of eggs, dairy products, and breakfast. The proportions of eating sweet foods, drinking sugary beverages, dining out, and eating Western fast food also declined. All changes were statistically significant (*p* < 0.05) except for the changes in vegetables and fried foods (Table 2).

The intake of fruits increased significantly after the system was launched, and the median intake increased from 0.00 (P25–P75: 0.00–107.90) g to 100.00 (P25–P75: 0.00–195.00). The proportion of people who reached the recommended values in the nutrition guidelines for students’ meals increased from 8.7% to 12.1%. The median soybean nuts intake level was 0.00 (P25–P75: 0.00–10.93) g, showing the intake of soybean nuts increasing significantly after the system usage (*p* < 0.05). The proportion of people who reached the recommended values in the nutrition guidelines for students’ meals increased from 19.4% to 32.8%. The median cereals and tubers intake level was 243.00 (P25–P75: 152.62–385.89) g, showing the intake of soybean nuts increasing significantly after the system usage (*p* < 0.05). The proportion of people who reached the recommended values in the nutrition guidelines for students’ meals increased from 32.0% to 51.7%, as shown in Table 3.

### 3.3. Comparison of Students’ Dietary Dairy, Vegetable, and Fruit Intakes Before and After the Digital Nudge Intervention According to Demographic Characteristics

This study mainly focuses on teenagers, whose dietary issues primarily involve insufficient intake of dairy products and fruits, and vegetables. Therefore, our study also involved relevant survey research on this matter. In the initial surveys of the study, it was found that girls’ intake of vegetables and fruits was generally higher than that of boys. With the use of smart canteens, among the male adolescent group surveyed, their fruit intake significantly increased (*p* < 0.05), with the median intake growing from 0.00 (P25–P75: 0.00–100.00) g to 100 (P25–P75: 0.00–212.50) g. In terms of household food consumption patterns, 30–40% of families saw a significant increase in fruit consumption (*p* < 0.05) after using smart canteens, with the median increasing from 0.10 (P25–P75: 0.00–112.50) g to 100 (P25–P75: 0.00–200.00) g. Additionally, there was a statistically significant difference in the data before and after the use of smart dining halls among adolescents whose primary caregivers had an educational level of university or above (*p* < 0.05). As shown in Table 4. In terms of dairy product consumption, male students’ intake is generally higher than females, but both fall short of the recommended intake standards. After the launch of the smart canteen, regardless of the proportion of food expenditure to total household expenditure or the different levels of education of the primary caregivers, the median consumption of dairy products has shown an upward trend. See Table 5 for details.

### 3.4. Comparison of Students’ Dietary Nutrient Intakes Before and After the Digital Nudge Intervention in Smart School Canteen

After analyzing the nutrient intake of students’ meals, it was found that calcium intake showed an increasing trend after the system was launched (*p* = 0.093, *p* < 0.1), with the median intake rising from 683.00 (P25–P75: 518.00–980.21) mg to 804.11 (P25–P75: 468.89–1166.74) mg, reaching the recommended value in the student meal nutrition guidelines from 39.3% to 50.9%. There was no statistically significant difference in the intake of other nutrients before and after the system launch. A detailed analysis of the overall intake can be found in Table 6.

To further investigate the dietary issues among adolescents (insufficient intake of dairy products and fruits, and vegetables), we conducted additional surveys on the intake of two major nutrients: calcium and vitamin C. In the baseline survey, we found that the median intake of calcium and vitamin C nutrients for males was 692.00 mg and 154.02 mg, respectively, both higher than those for females. Notably, in families where food expenditure accounted for less than 30% of total household spending, the intake of calcium and vitamin C was higher than in other family income levels, with median intakes of 801.03 (P25–P75: 579.06–1027.23) mg and 136.80 (P25–P75: 54.72–238.48) mg, respectively. Additionally, regarding the educational level of the primary caregivers, families where the caregivers had a university education or above had a median calcium intake of 718.88 (P25–P75: 522.76–980.21) mg, while the median vitamin C intake was 126.94 mg (P25–P75: 61.96–251.75) mg. Detailed data can be found in Table 7.

## 4. Discussion

This study implemented a smart canteen system and multi-strategy digital nudge interventions in a public high school in Shenyang, China, and found that students’ healthy food consumption behaviors significantly improved after the intervention. Specifically, the daily intake frequency of whole grains, fruits, seafood, and soy products significantly increased (*p* < 0.05), while unhealthy dietary behaviors such as sweet foods, sugary drinks, dining out, and fast food consumption significantly decreased. In terms of nutrient intake, the average daily calcium intake increased from 669.74 mg at baseline to 811.33 mg after the intervention (*p* < 0.05), and the appropriate intake rate increased from 38.8% to 51.7%, becoming the most significant improvement indicator. However, the intake of other key nutrients, such as vitamin C, increased slightly but did not reach statistical significance. In addition, the fruit intake of male students significantly increased after the intervention, while the change in female students was not significant, suggesting that gender differences may affect the intervention effect.

This study primarily employed digital nudging interventions, which can significantly promote the selection of healthy foods. This responded to our first research hypothesis. This outcome is consistent with several international studies. For instance, the Australian Online Canteen study demonstrated that the choice architecture intervention provided by online canteens can encourage high school students to choose healthy diets [19]. This study further validated that the intervention effect of digital environments in optimizing adolescent dietary decision-making is more significant. Other traditional nudging methods mainly rely on the physical environment or interpersonal interactions. For example, a research team in the United States implemented the Smarter Lunchrooms project on campus, placing vegetables in prominent positions on the canteen line or repeating their placement and giving them creative names. Through RCT research analysis, it is known that the selection rates of vegetables, fruits, and milk have all increased (*p* < 0.05) [20]. In summary, the difference between digital nudging and traditional nudging models is not significant, mainly because digital nudging is not limited by time or location, and due to the flexibility and real-time feedback of digital technology, a mixed nudging model using various nudging methods can be employed.

This study, respectively, explored the food consumption frequency and dietary intake in school meals through the application of a digital nudge intervention strategy. The findings suggested that the ordering system of a digital nudge intervention can improve the daily consumption proportion of healthy foods, such as whole grains, aquatic products, and soy products, as indicated by the investigation of food consumption frequency. Also, it was found that the intake of grains, potatoes, and nuts and seeds has significantly increased. Similarly, this responded to our second research hypothesis. In summary, this study indicates that a smart canteen can improve students’ dietary choices and intake, which is similar to the findings of a study in the United States. That study found that through smart canteen interventions, students’ average choice of whole grains increased by about 0.44 servings, the choice of refined grains decreased by about 0.33 servings, and the choice of fruits increased by about 0.39 servings [21]. A meta-study indicates that nudging can influence people’s diet, being more effective in reducing unhealthy eating than in increasing healthy eating [22]. In a systematic review on the effects of nudging on students’ dietary behavior [5], 79% of the studies showed that nudging had a positive effect on food choices. From a population perspective, the design of intelligent dining halls for primary and secondary school students can effectively affect the intake of energy, saturated fat, sugar, and sodium, producing positive effects [11,12,23]. Even when the population is switched to adults, there are similar conclusions. A longitudinal study in a large hospital cafeteria found that a two-year choice architecture intervention, including traffic light labeling, product placement, and promotions, increased the sales of “healthy” items by 5% and decreased the sales of unhealthy items by 3% (*p* < 0.001) [24].

Furthermore, the investigation also found an issue where students’ intake of fruits and vegetables, as well as dairy products, is severely insufficient, significantly less than the recommended intake levels mentioned in the “Student Meal Nutrition Guide” [18]. This is also a widespread problem, as a survey conducted in Beijing on 3rd to 12th grade school students also found that the intake of fruits and vegetables in this area is generally insufficient [25]. Therefore, we further investigated students’ intake of dairy products and fruits, and vegetables. Accordingly, dairy and dairy products are an important category of food, rich in calcium and conducive to absorption. The calcium content in every 100 mL of fresh milk is about 100~110 mg [18]. At the same time, fruits and vegetables contain a large amount of vitamin C [18]. Thus, we also conducted research on the corresponding nutrients. All in all, the system improved dairy products and fruits, and vegetables as well as the corresponding nutrients, but the effects vary for different foods and nutrients, and the effectiveness also differs among different demographic characteristics. Thus, we also conducted a survey and research on the corresponding nutrients. The study found that gender factors and economic factors (the proportion of food in total household expenditure) affect students’ consumption of dairy products, fruits and vegetables, and nutrients. These findings are consistent with previous conclusions. A meta-analysis of global school students’ health surveys shows that fruit consumption is particularly rare in South and East Asia [26]. Data from the World Health Organization’s Regional Office for Europe’s quadrennial survey of health behaviors in school-aged children (HBSC) indicate that at least half of adolescents do not eat fruit daily, suggesting that adolescents worldwide are insufficient in their intake of fruits and vegetables. In terms of fruit consumption, girls have a higher consumption rate (47% compared to 32% for boys), and there is a social gradient in daily fruit consumption, with adolescents from affluent families having a higher consumption level. In Kazakhstan, the difference between high-income and low-income groups is the greatest for both boys and girls (23 and 21 percentage points, respectively), and the same is true in the United Kingdom (25 percentage points for both boys and girls) [27]. Among children and adolescents, the proportion of those meeting the recommended daily intake of 300 g of dairy products is low. Although intake has increased from 1991 to 2018, there is a significant gap compared to the recommended levels. Domestic data of China indicates that the compliance rate for dairy product intake among children and adolescents aged 6 to 17 is only 2.86% [28]. Factors such as economic development, cultural level, and dietary traditions may affect the formation of sustained dairy consumption habits [29,30]. The production and supply capacity of dairy products, as well as the diversity of types, are also key factors.

These research findings suggest that the smart canteen intervention with embedded digital nudge interventions has potential value in improving the nutritional quality of students’ food choices in the short term. At the same time, the merits and limitations of this study’s results should be considered. It is one of the few studies in China that evaluates the impact of digital nudging on smart canteens regarding food choices.

There are some limitations that should be noted in our study. Firstly, the research subjects are relatively single. Due to the difficulty in coordination during actual research, the subjects only include students from two grades in one school. In addition, the effect time after the ordering system is utilized is relatively short, only covering 3 months, which makes it impossible to explore the long-term effects of the smart school canteen system based on digital nudging on students’ dietary habits and health outcomes. In the future, the next step of research can expand the scope of the subjects, which will be more conducive to understanding the impact of the smart canteen system based on digital nudging on students’ dietary choices. At the same time, it can also be extended to at least 15 months to study the long-term effects of the system [19].

## 5. Conclusions

Our findings support the assertion that the smart canteen system with embedded digital nudge interventions plays an important role in shaping students’ dietary choices. The intake of food and nutrients has both improved, with a significant increase in the consumption of foods such as cereals, potatoes, fruits, soybeans/nuts, and nutrients like calcium, and the proportion reaching the recommended values has also increased significantly. For a long time, there has been a lack of research on the application of embedded digital nudge interventions in smart canteens in China. This study provides empirical evidence for policymakers, but further research is still needed to deepen the understanding of this field. The World Health Organization has emphasized that using online food delivery systems to achieve public health benefits is an important direction at present [31]. Therefore, how to utilize current technological capabilities to build an intelligent canteen system suitable for students, in order to enhance the quality of meals and student diets, construct a good dining environment, improve the dining experience, promote nutrition education, and thereby truly enhance the nutritional intake and physical fitness of children and adolescents, and increase satisfaction among families, schools, and students, is a topic worth discussing for schools and relevant personnel.

## Figures and Tables

**Table 1:** Demographic characteristics of participants at baseline.

Demographic Characteristics	*n*	Percentage (%)
Gender		
Male	244	48.6
Female	258	51.4
Grade		
10th	278	55.4
11th	224	44.6
Household registration ^a^		
Rural	114	27.1
Urban	306	72.9
Proportion of household food expenditure ^a^		
<30%	96	23.7
30–40%	192	47.4
40–50%	61	15.1
>50%	56	13.8
Principal caregiver ^a^		
Mother	371	81.4
Father	69	15.1
Other	16	3.5
Principal caregiver’s education level ^a^		
High school education or below	225	54.5
Junior college or above	188	45.5
	*n* = 502	100

^a^ Data missing.

**Table 2:** Comparison of students’ food consumption behaviors before and after digital nudge intervention in the smart school canteen.

Food Consumption During the Past 7 Days	Baseline *n* (%)	Post-Intervention *n* (%)	*p*
Coarse grains (daily)	88 (18.60)	49 (23.90)	0.017 ^a^
Vegetables (daily)	267 (71.84)	168 (72.17)	0.172
Fruits (daily)	209 (41.69)	132 (54.87)	<0.001 ^a^
Seafood (daily)	294 (74.04)	199 (82.95)	<0.001 ^a^
Eggs (daily)	177 (47.81)	110 (46.02)	<0.001 ^a^
Dairy products (daily)	254 (68.50)	138 (57.34)	<0.001 ^a^
Soy products (daily)	64 (17.36)	51 (21.24)	<0.001 ^a^
Breakfast (daily)	299 (81.74)	187 (77.91)	<0.001 ^a^
Sweets (daily)	62 (16.71)	44 (18.32)	0.033 ^a^
Sugary drinks (daily)	106 (28.78)	57 (23.84)	0.015 ^a^
Fried foods (daily)	79 (21.47)	48 (19.97)	0.247
Eating out (daily)	181 (49.19)	94 (39.29)	<0.001 ^a^
Fast food (daily)	224 (60.26)	124 (51.76)	<0.001 ^a^

^a^ *p* < 0.05.

**Table 3:** Comparison of students’ food intakes before and after the digital nudge intervention in the smart school canteen.

Foods	Baseline Intakes			Dietary Intakes After Intervention		
	Mean (g/d)	P50 (P25–P75) (g/d)	Sufficient/Appropriate Intake Rate ^b^ (%)	Mean (g/d)	P50 (P25–P75) (g/d)	Sufficient/Appropriate Intake Rate ^b^ (%)
Cereals and tubers	281.67	243.00 (152.62–385.89)	32.0	387.63	351.25 (290.77–475.62) ^a^	51.7
Vegetables	250.49	200.00 (100.00–383.38)	15.0	266.65	213.22 (111.20–387.56)	16.4
Fruits	89.27	0.00 (0.00–107.90)	8.7	106.54	100.00 (0.00–195.00) ^a^	12.1
Livestock and poultry meat	136.67	100.00 (58.52–200)	74.8	156.09	116.90 (74.86–202.06)	77.6
Aquatic products	28.60	0.00 (0.00–42.48)	22.4	31.65	0.00 (0.00–58.65)	29.6
Eggs	63.39	50.32 (0.07–100.00)	45.1	71.28	98.20 (9.58–100.00)	52.6
Dairy products	87.56	100.00 (0.00–107.00)	7.8	88.44	100.00 (0.00–111.55)	7.8
Soybeans and nuts	21.93	0.00 (0.00–10.93)	19.4	40.96	7.00 (0.00–77.15)	32.8

^a^ *p* < 0.05, *p-value* of food intakes (percentile) compared with baseline. ^b^ The food intakes were recognized as sufficient or appropriate according to the Chinese nutrition guidelines of school meal (WS/T 554) [18].

**Table 4:** Comparison of students’ dietary vegetable and fruit intakes before and after the digital nudge intervention according to demographic characteristics.

Demographic Characteristics	Dietary Vegetable Intake, P50 (P25–P75), g/d			Dietary Fruit Intake, P50 (P25–P75), g/d		
	Baseline	Post-Intervention	*p*	Baseline	Post-Intervention	*p*
Gender						
Male	190.10 (97.00–337.60)	205.10 (117.80–369.83)	0.186	0.00 (0.00–100.00)	100.00 (0.00–212.50)	0.006 ^a^
Female	213.60 (117.20–400.00)	221.25 (100.00–400.00)	0.834	50.00 (0.00–200.00)	100.00 (0.00–150.00)	0.846
Proportion of household food expenditure						
<30%	181.50 (75.78–423.50)	206.80 (105.30–349.30)	0.967	0.00 (0.00–150.00)	100.00 (0.00–150.00)	0.315
30–40%	207.30 (117.06–400.00)	251.45 (158.65–400.00)	0.390	0.10 (0.00–112.50)	100.00 (0.00–200.00)	0.028 ^a^
40–50%	183.70 (107.65–302.35)	203.70 (90.20–400.00)	0.925	0.00 (0.00–100.00)	50.00 (0.00–200.00)	0.454
>50%	190.73 (100.00–346.88)	196.10 (157.10–475.00)	0.122	0.00 (0.00–125.00)	100.00 (0.00–195.00)	0.540
Principal caregiver’s education level						
High school education or below	200.00 (100.00–349.30)	200.00 (107.90–400.00)	0.878	19.80 (0.00–150.00)	100.00 (0.00–150.00)	0.177
Junior college or above	200.00 (100.50–391.70)	251.45 (100.00–397.90)	0.522	0.00 (0.00–100.00)	100.00 (0.00–200.00)	0.093 ^b^

^a^ *p* < 0.05 compared with baseline. ^b^ *p* < 0.1 compared with baseline.

**Table 5:** Comparison of students’ dietary dairy intake before and after the digital nudge intervention according to demographic characteristics.

Demographic Characteristics	Dietary Dairy Intake, P50(P25–P75), g/d		
	Baseline	Post-Intervention	*p*
Gender			
Male	100.00 (0.00–100.00)	100.00 (0.00–150.00)	0.679
Female	50.00 (0.00–150.00)	50.00 (0.00–100.00)	0.441
Proportion of household food expenditure			
<30%	50.00 (0.00–200.00)	50.50 (0.00–125.00)	0.892
30–40%	100.00 (0.00–100.00)	100.00 (0.00–141.88)	0.485
40–50%	50.00 (0.00–100.00)	100.00 (0.00–100.00)	0.740
>50%	100.00 (0.00–107.50)	100.00 (0.00–100.00)	0.535
Principal caregiver’s education level			
High school education or below	50.00 (0.00–100.00)	50.00 (0.00–115.40)	0.779
Junior college or above	100.00 (0.00–128.75)	100.00 (0.00–133.75)	0.421

**Table 6:** Comparison of students’ daily nutrient intakes before and after the digital nudge intervention in the smart school canteen.

Nutrients	Baseline Intakes		Dietary Intakes After Intervention		*p*
	P50 (P25–P75)	Sufficient/Appropriate Intake Rate ^b^ (%)	P50 (P25–P75)	Sufficient/Appropriate Intake Rate ^b^ (%)	
Protein (g/d)	66.71 (52.41–86.17)	62.5	69.55 (47.94–90.83)	71.2	0.992
Fat (%E)	26.12 (20.75–32.63)	79.1	27.73 (22.84–34.03)	87.5	0.148
Carbohydrate (%E)	52.90 (45.91–59.50)	60.2	52.19 (42.98–58.97)	57.1	0.398
Calcium (mg/d)	683.00 (518.00–980.21)	39.3	804.11 (468.89–1166.74)	50.9	0.093 ^a^
Iron (mg/d)	25.44 (18.42–32.60)	90.6	25.85 (20.16–35.61)	91.1	0.377
Zinc (mg/d)	15.22 (10.52–20.02)	86.9	15.02 (10.68–21.38)	92.9	0.550
Vitamin A (μg RAE/d)	662.91 (375.52–1008.00)	62.8	743.68 (353.28–1068.00)	66.1	0.716
Vitamin B1 (mg/d)	1.14 (0.85–1.54)	40.8	1.13 (0.88–1.66)	42.0	0.746
Vitamin B2 (mg/d)	0.72 (0.53–0.96)	16.8	0.77 (0.53–1.03)	19.6	0.384
Vitamin C (mg/d)	140.00 (68.06–258.22)	67.0	159.63 (73.77–280.17)	74.1	0.192

^a^ *p* < 0.1, *p-value* of nutrient intakes (percentile) compared with baseline. ^b^ The dietary intakes of protein, calcium, iron, zinc, and vitamins A, B1, B2, and C, exceeding the recommended nutrient intakes (RNI), were recognized as sufficient, and energy percent of fat and carbohydrate were considered as appropriate when they fell within acceptable macronutrient dietary range (AMDR), according to Chinese dietary reference intakes (DRIs) [18].

**Table 7:** Comparison of students’ dietary calcium and vitamin C intakes before and after the digital nudge intervention according to demographic characteristics.

Demographic Characteristics	Dietary Calcium Intake, P50 (P25–P75), mg/d			Dietary Vitamin C Intake, P50 (P25–P75), mg/d		
	Baseline	Post-Intervention	*p*	Baseline	Post-Intervention	*p*
Gender						
Male	669.10 (540.00–908.83)	836.24 (512.73–1210.84)	0.015 ^a^	112.62 (59.71–229.21)	166.80 (79.07–320.75)	0.023 ^a^
Female	692.00 (481.58–1014.82)	766.73 (467.88–1084.00)	0.379	154.02 (73.52–285.07)	155.10 (69.62–259.64)	0.611
Proportion of household food expenditure						
<30%	801.03 (579.06–1027.23)	869.68 (472.93–1397.76)	0.339	136.80 (54.73–238.48)	151.52 (58.92–333.65)	0.514
30–40%	682.29 (514.00–952.00)	774.98 (459.90–1069.36)	0.312	135.46 (62.43–290.00)	160.57 (86.05–294.29)	0.259
40–50%	648.00 (445.19–782.60)	779.35 (381.18–1194.42)	0.081 ^b^	133.57 (78.34–326.74)	135.33 (71.48–275.91)	0.948
>50%	711.13 (331.25–1068.01)	753.03 (528.72–1019.72)	0.708	136.79 (56.23–272.68)	158.99 (39.99–184.28)	0.364
Principal caregiver’s education level						
High school education or below	648.00 (481.58–890.80)	808.78 (552.14–1215.81)	0.054 ^b^	154.02 (64.06–268.97)	173.69 (97.47–346.66)	0.155
Junior college or above	718.88 (522.76–980.21)	746.59 (455.37–1089.41)	0.307	126.94 (61.96–251.75)	126.99 (53.78–276.08)	0.556

^a^ *p* < 0.05, compared with baseline. ^b^ *p* < 0.1, compared with baseline.

## Data Availability

The data sets generated or analyzed during this study are available from the corresponding author on reasonable request. The data sets are not publicly available due to ethical considerations.

## References

[1] Jones M., Lynch K.T., Kass A.E., Burrows A., Williams J., Wilfley D.E., Taylor C.B. (2014). Healthy weight regulation and eating disorder prevention in high school students: A universal and targeted Web-based intervention. J. Med. Internet Res..

[2] World Health Organization (2022). Nudges to Promote Healthy Eating in Schools: Policy Brief.

[3] Jia P., Li M., Xue H., Lu L., Xu F., Wang Y. (2017). School environment and policies, child eating behavior and overweight/obesity in urban China: The childhood obesity study in China megacities. Int. J. Obes..

[4] Global Child Nutrition Foundation (2021). School Meal Programs Around the World: Results from the 2021 Global Survey of School Meal Programs.

[5] Metcalfe J.J., Ellison B., Hamdi N., Richardson R., Prescott M.P. (2020). A systematic review of school meal nudge interventions to improve youth food behaviors. Int. J. Behav. Nutr. Phys. Act..

[6] Downs S., Demmler K.M. (2020). Food environment interventions targeting children and adolescents: A scoping review. Glob. Food Secur..

[7] Wyse R., Delaney T., Stacey F., Lecathelinais C., Ball K., Zoetemeyer R., Lamont H., Sutherland R., Nathan N., Wiggers J.H. (2021). Long-term effectiveness of a multistrategy behavioral intervention to increase the nutritional quality of primary school students’ online lunch orders: 18-month follow-up of the Click & Crunch cluster randomized controlled trial. J. Med. Internet Res..

[8] Granheim S.I., Løvhaug A.L., Terragni L., Torheim L.E., Thurston M. (2022). Mapping the digital food environment: A systematic scoping review. Obes. Rev..

[9] Özdemir Ş. (2019). Digital nudges and dark patterns: The angels and the archfiends of digital communication. Digit. Sch. Humanit..

[10] Leonard A., Delaney T., Seward K., Zoetemeyer R., Lamont H., Sutherland R., Reilly K., Lecathelinais C., Wyse R. (2021). Investigating differences between traditional (paper bag) ordering and online ordering from primary school canteens: A cross-sectional study comparing menu, usage and lunch order characteristics. Public Health Nutr..

[11] Delaney T., Wyse R., Yoong S.L., Sutherland R., Wiggers J., Ball K., Campbell K., Rissel C., Lecathelinais C., Wolfenden L. (2017). Cluster randomized controlled trial of a consumer behavior intervention to improve healthy food purchases from online canteens. Am. J. Clin. Nutr..

[12] Delaney T., Jackson J., Lecathelinais C., Yoong S.L., Wolfenden L., Sutherland R., Webb E., Wyse R. (2023). Exploratory analysis of a cluster randomized controlled trial of a multi-strategy intervention delivered via online canteens on improving the nutritional quality of primary school students’ pre-ordered foods & drinks at recess. Appetite.

[13] World Food Programme (2020). State of School Feeding Worldwide 2020.

[14] Vellinga R.E., Eykelenboom M., Olthof M.R., Steenhuis I.H.M., de Jonge R., Temme E.H.M. (2022). Less meat in the shopping basket: The effect on meat purchases of higher prices, an information nudge and the combination: A randomized controlled trial. BMC Public Health.

[15] Ministry of Education of the People’s Republic of China (2020). Action Plan for the “Preventing Food Waste and Cultivating Saving Habits” in the Education System. http://www.moe.gov.cn/srcsite/A03/s7050/202009/t20200915_488025.html.

[16] Chinese Nutrition Society Chinese Dietary Guidelines for School-Age Children. 19 May 2022. https://www.cnsoc.org/dGuideline/122510200.html.

[17] The National Health and Family Planning Commission of the People’s Republic of China Student Meal Nutrition Guidelines. 1 August 2017. http://www.nhc.gov.cn/ewebeditor/uploadfile/2017/08/01/20170801100101818.pdf.

[18] Chinese Nutrition Society (2023). Chinese Residents’ Dietary Nutrient Reference Intakes: 2023 Edition.

[19] Delaney T., Jackson J., Lecathelinais C., Clinton-McHarg T., Lamont H., Yoong S.L., Wolfenden L., Sutherland R., Wyse R. (2024). Long-term effectiveness of a multi-strategy choice architecture intervention in increasing healthy food choices of high-school students from online canteens (Click & Crunch High Schools): Cluster randomized controlled trial. J. Med. Internet Res..

[20] Greene K.N., Gabrielyan G., Just D.R., Wansink B. (2017). Fruit-promoting smarter lunchrooms interventions: Results from a cluster RCT. Am. J. Prev. Med..

[21] Hubbard K.L., Bandini L.G., Folta S.C., Wansink B., Eliasziw M., Must A. (2015). Impact of a Smarter Lunchroom intervention on food selection and consumption among adolescents and young adults with intellectual and developmental disabilities in a residential school setting. Public Health Nutr..

[22] Cadario R., Chandon P. (2020). Which healthy eating nudges work best? A meta-analysis of field experiments. Mark. Sci..

[23] Delaney T., Yoong S.L., Lamont H., Lecathelinais C., Wolfenden L., Clinton-McHarg T., Sutherland R., Wyse R. (2022). The efficacy of a multi-strategy choice architecture intervention on improving the nutritional quality of high school students’ lunch purchases from online canteens (Click & Crunch High Schools): A cluster randomized controlled trial. Int. J. Behav. Nutr. Phys. Act..

[24] Thorndike A.N., Riis J., Sonnenberg L.M., Levy D.E. (2014). Traffic-light labels and choice architecture: Promoting healthy food choices. Am. J. Prev. Med..

[25] Wu X., Yu Y., He H., Yu X., Guo D., Zhu W. (2024). Individual and family factors correlated with children’s fruit consumption. Front. Public Health.

[26] Beal T., Morris S.S., Tumilowicz A. (2019). Global patterns of adolescent fruit, vegetable, carbonated soft drink, and fast-food consumption: A meta-analysis of global school-based student health surveys. Food Nutr. Bull..

[27] Költő A., de Looze M., Jåstad A., Nealon Lennox O., Currie D., Gabhainn S.N. (2024). A Focus on Adolescent Sexual Health in Europe, Central Asia and Canada: Health Behaviour in School-Aged Children International Report from the 2021/2022 Survey.

[28] Jia S., Hongyun F., Dongmei Y., Liyun Z., Lahaohong J., Qiyayi G., Li H. (2022). Analysis of milk product intake by Chinese children and adolescents aged 6–17 years old. Chin. Food Nutr..

[29] Zhang J., Wang D., Eldridge A.L., Huang F., Ouyang Y., Wang H., Zhang B. (2017). Urban-rural disparities in energy intake and contribution of fat and animal source foods in Chinese children aged 4–17 years. Nutrients.

[30] Xu P.P., Yang T.T., Xu J., Li L., Cao W., Gan Q., Hu X.Q., Pan H., Zhao W.H., Zhang Q. (2019). Dairy consumption and associations with nutritional status of Chinese children and adolescents. Biomed. Env. Sci..

[31] World Health Organization (2021). Slide to Order: A Food Systems Approach to Meals Delivery Apps: WHO European Office for the Prevention and Control of Non-Communicable Diseases.

